# NAT10 Promotes Prostate Cancer Growth and Metastasis by Acetylating mRNAs of HMGA1 and KRT8

**DOI:** 10.1002/advs.202310131

**Published:** 2024-06-23

**Authors:** Kang‐Jing Li, Yaying Hong, Yu‐Zhong Yu, Zhiyue Xie, Dao‐Jun Lv, Chong Wang, Tao Xie, Hong Chen, Zhe‐Sheng Chen, Jianwen Zeng, Shan‐Chao Zhao

**Affiliations:** ^1^ Department of Urology Nanfang Hospital Southern Medical University Guangzhou 510515 China; ^2^ Department of Urology Affiliated Qingyuan Hospital Guangzhou Medical University Qingyuan People's Hospital Qingyuan 511518 China; ^3^ Department of Urology The Third Affiliated Hospital of Guangzhou Medical University Guangzhou 510150 China; ^4^ Luoyang Key Laboratory of Organic Functional Molecules College of Food and Drug Luoyang Normal University Luoyang Henan 471934 P. R. China; ^5^ Department of Pharmaceutical Sciences College of Pharmacy and Health Sciences St. John's University Queens NY 11439 USA; ^6^ Department of Urology The Fifth Affiliated Hospital Southern Medical University Guangzhou 510900 China; ^7^ Department of Urology The Third Affiliated Hospital of Southern Medical University Guangzhou 510500 China

**Keywords:** ac4C, HMGA1, KRT8, NAT10, prostate cancer

## Abstract

N4‐acetylcytidine (ac4C) is essential for the development and migration of tumor cells. According to earlier research, N‐acetyltransferase 10 (NAT10) can increase messenger RNAs (mRNAs) stability by catalyzing the synthesis of ac4C. However, little is known about NAT10 expression and its role in the acetylation modifications in prostate cancer (PCa). Thus, the biological function of NAT10 in PCa is investigated in this study. Compared to paraneoplastic tissues, the expression of NAT10 is significantly higher in PCa. The NAT10 expression is strongly correlated with the pathological grade, clinical stage, Gleason score, T‐stage, and N‐stage of PCa. NAT10 has the ability to advance the cell cycle and the epithelial‐mesenchymal transition (EMT), both of which raise the malignancy of tumor cells. Mechanistically, NAT10 enhance the stability of high mobility group AT‐hook 1 (HMGA1) by acetylating its mRNA, thereby promoting cell cycle progression to improve cell proliferation. In addition, NAT10 improve the stability of Keratin 8 (KRT8) by acetylating its mRNA, which promotes the progression of EMT to improve cell migration. This findings provide a potential prognostic or therapeutic target for PCa.

## Graphical abstract

Increased NAT10 expression accounted for inferior clinicopathological characteristics in patients with PCa. NAT10 can promote prostate cancer growth and metastasis by catalyzing the formation of ac4C. Mechanistically, NAT10 enhance the stability of HMGA1 by acetylating its mRNA, thereby promoting cell cycle progression to improve cell proliferation. In addition, NAT10 improve the stability of KRT8 by acetylating its mRNA, which promotes the progression of epithelial‐mesenchymal transition to improve cell migration.

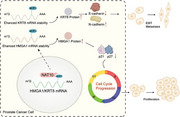

## 1 Introduction

Prostate cancer (PCa) is an increasingly prevalent disease that has become one of the world's leading causes of cancer‐related mortality for men in recent years.^[^ ^1^, ^2^ ^]^ Most patients experience remission from tumor progression after androgen deprivation therapy. However, after 18 to 24 months of treatment, patients frequently develop castration‐resistant PCa, which makes follow‐up treatment challenging and significantly worsens the patients’ prognosis.^[^ ^3^, ^4^ ^]^

Epigenetic modification of ribonucleic acid (RNA) has become a popular research topic in recent years. Epigenetic modification can regulate downstream protein synthesis by affecting RNA production, transport, metabolism, translation efficiency, and so on. N6‐methyladenosine (m6A), 5‐formylcytidine (f5C), 7‐methylguanosine (m7G), and N4‐acetylcytidine (ac4C) are typical RNA modifications.^[^ ^5^, ^6^, ^7^, ^8^ ^]^ Following m7G, m6A, and f5C, the first acetylated nucleosides to be identified, ac4C was also found to be present in large quantities on messenger RNAs (mRNAs).^[^ ^9^, ^10^ ^]^

N‐acetyltransferase 10 (NAT10), a crucial member of the Gcn5‐related N‐acetyltransferase family, has a molecular weight of 115 KD and 1025 amino acids. It is a protein acetyltransferase that acetylates a variety of target proteins, such as histones, a‐tubulin, and MORC family CW‐type zinc finger 2 (MORC2).^[^ ^11^, ^12^, ^13^ ^]^ Initially detected on transfer RNA (tRNA) and ribosomal RNA (rRNA),^[^ ^14^, ^15^ ^]^ ac4C has also been found to be widespread in human and yeast mRNA in recent years.^[^ ^9^, ^10^ ^]^ NAT10, the only mRNA acetyltransferase discovered so far, can catalyze a whole generation of ac4C modifications of mRNA, affecting the translational efficiency or stability of downstream target genes and thus influencing the maturation of germ cells, bone reconstruction, tumor development, and systemic lupus erythematosus, among other diseases.^[^ ^16^, ^17^, ^18^, ^19^, ^20^ ^]^ NAT10 mediated ac4C acetylation of murine double minute 2 (MDM2) transcripts and subsequently stabilized MDM2 mRNA, leading to its upregulation and p53 downregulation, thereby promoting gastric carcinogenesis.^[^ ^21^ ^]^ NAT10 is involved in the regulation of cancer cell proliferation, migration, invasion, survival and stem cell‐like properties through its involvement in ac4C modification of B cell CLL/lymphoma 9 like (BCL9L), SRY‐box transcription factor 4 (SOX4), and AKT serine/threonine kinase 1 (AKT1) in bladder cancer.^[^ ^22^ ^]^ However, there is a dearth of research on the biological role of NAT10‐catalyzed ac4C in the development of PCa. It is highly likely that it may be a potential new target for the identification and treatment of PCa.

Therefore, in this study, the endogenous expression of NAT10 and its correlation with clinical data from PCa patients were examined by mining information from public databases and clinical tissue samples. In addition, we clarified by further mechanistic experiments that NAT10 might lead to PCa cell growth and metastasis by catalyzing the ac4C acetylation of downstream high mobility group AT‐hook 1 (HMGA1) and keratin 8 (KRT8) mRNAs and subsequently stabilizing their mRNAs, leading to cell cycle progression and epithelial‐mesenchymal transition (EMT).

## 2 Results

### 2.1 NAT10 is Upregulated in PCa Tissues and Correlated with Clinicopathological Parameters

**Figure 1:**
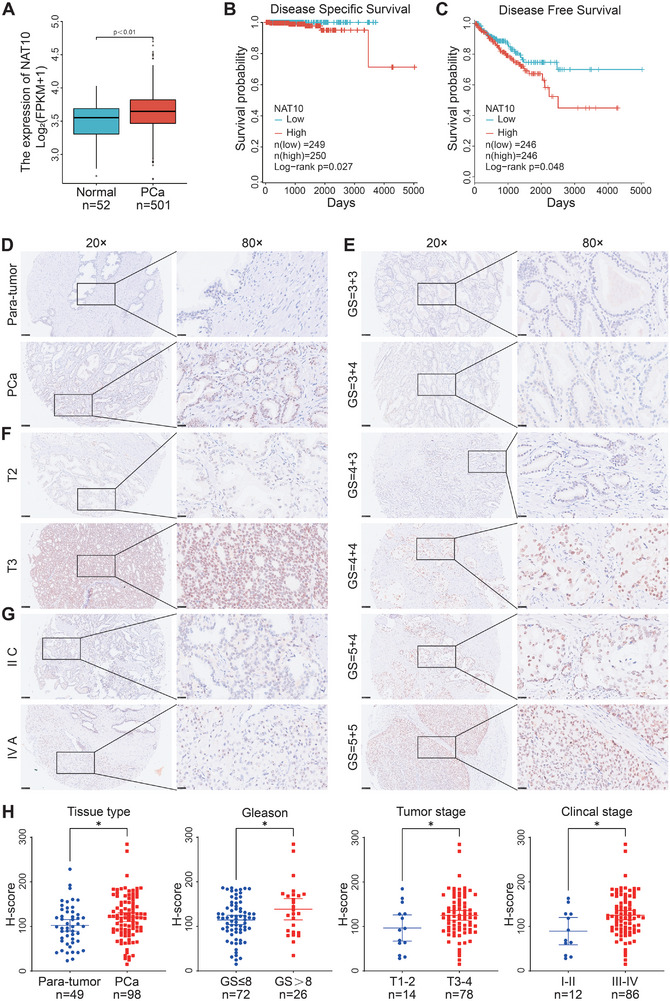
NAT10 expression is upregulated in PCa tissues and correlated with clinicopathological parameters. A) NAT10 is overexpressed in TCGA PCa data. B,C) The highly expressed group had low disease‐specific and disease‐free survival rate, according to a survival analysis of NAT10 (TCGA Prostate adenocarcinoma). D) Comparison of NAT10 expression in para‐tumor and PCa samples in the TMA. E–G) The TMA revealed a connection between NAT10 expression and clinical characteristics in patients with PCa. H) Graphical illustration of statistical results of H‐score. Scale bar = 25 and 100 µm.

To determine the expression of NAT10 in PCa, we analyzed the expression of NAT10 in data from The Cancer Genome Atlas (TCGA) database (https://www.xiantao.love/) using bioinformatics methods. Results showed that the expression level of NAT10 was significantly upregulated in PCa tissues compared to normal prostate tissues (**Figure** **1**A). In addition, we also divided PCa patients into two groups on whether they had high or low expression of NAT10, and analyzed their clinicopathological parameters. We found that the patients in high NAT10 expression group had significantly lower disease‐specific survival and disease‐free survival rates (Figure 1B,C). In addition, patients with higher NAT10 expression had significantly higher N‐stage and Gleason scores (**Table** **1**). To further analyze the correlation between the protein level of NAT10 and the clinicopathological features of PCa, we stained specimens in the tissue microarray (TMA) of prostate cancer for NAT10 and analyzed them according to the H‐score, as quantitative data of staining intensity. The results showed that the cancerous tissues had significantly higher NAT10 protein levels than adjacent normal tissues, and patients with high NAT10 levels had higher T‐stages, Gleason scores, and clinical stages (Figure 1D–H). These data suggest that NAT10 may play an important role in the onset and development of PCa.

**Table 1:** Correlation between NAT10 expression and clinicopathological parameters in prostate cancer patients from TCGA database, n = 501.

Characteristics	NAT10 low expression	NAT10 high expression	P value
n	250	251	
T stage, n (%)			0.052 ^a^ ^)^
T1‐2	105 (21.3%)	84 (17.0%)	
T3‐4	142 (28.7%)	163 (33.0%)	
N stage, n (%)			0.033 ^a^ ^)^
N0	172 (40.2%)	176 (41.1%)	
N1	29 (6.8%)	51 (11.9%)	
M stage, n (%)			1.000 ^b^ ^)^
M0	230 (50.0%)	227 (49.3%)	
M1	2 (0.4%)	1 (0.2%)	
Gleason score, n (%)			0.040 ^a^ ^)^
6‐7	158 (31.5%)	136 (27.1%)	
8‐10	92 (18.4%)	115 (23.0%)	
Age, n (%)			0.683 ^a^ ^)^
≤60	110 (22.0%)	115 (23.0%)	
>60	140 (27.9%)	136 (27.1%)	

^a)^ P value is from χ2‐test;

^b)^ P value is from Yates's correction for continuity.

### 2.2 NAT10 Accelerates PCa Cell Proliferation In Vitro

**Figure 2:**
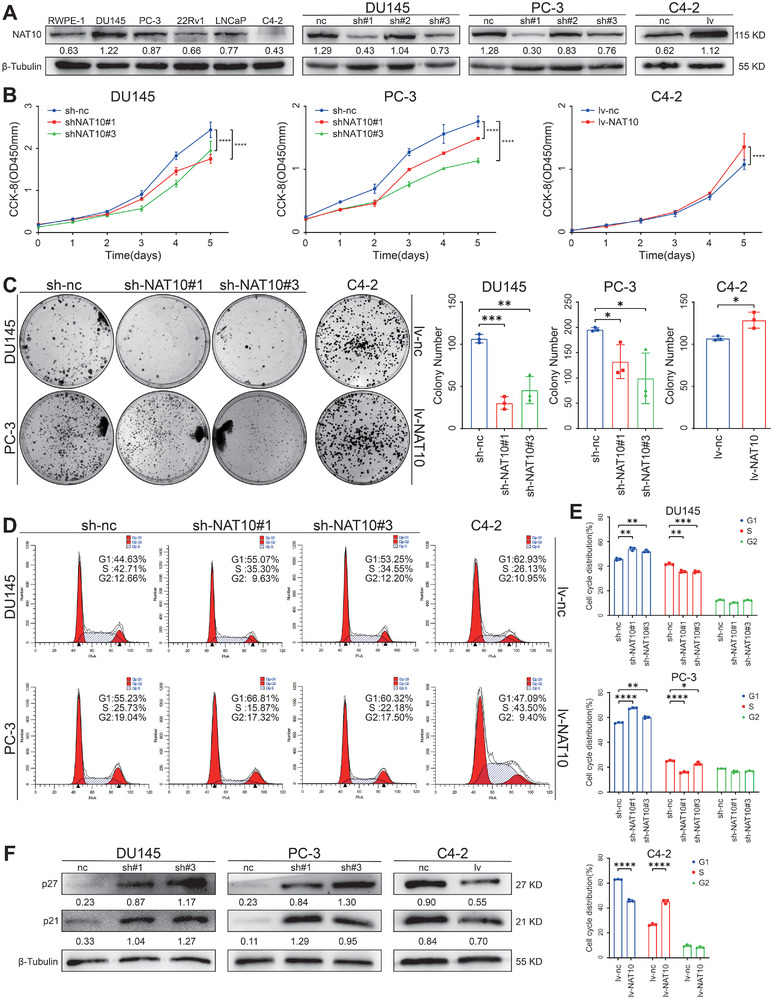
NAT10 accelerates PCa cell proliferation in vitro. A) The expression of NAT10 in PCa lines and efficiency of knockdown or overexpression of NAT10 were determined by the Western blot. B,C) PCa cell lines with NAT10 knockdown or overexpression were tested for proliferation using the CCK‐8 assay and the colony‐formation assay. D,E) Flow cytometric assays for the cell cycle profile of PCa cells stably transfected with NAT10. F) Expression of cell cycle‐related proteins was detected by the Western blot in PCa cell lines with or without knocking down or overexpressing NAT10.

To analyze the role of NAT10 in PCa, we first examined endogenous expression of NAT10 by Western blot in the immortalized normal prostate epithelial cell line RWPE‐1 and the PCa cell lines DU145, PC‐3, LNCaP, 22Rv1, and C4‐2. The expression levels of NAT10 in the RWPE‐1 cell line were lower than that of all other PCa cell lines except C4‐2. We selected DU145, PC‐3, and C4‐2 cells for subsequent studies based on the expression of the cell lines. We designed three short hairpin RNAs (shRNAs) targeting NAT10 to inhibit its expression in DU145 and PC‐3 cells. Following our assessment, we identified sh‐NAT10#1 and sh‐NAT10#3 as having significant inhibitory ability, and these were chosen for subsequent experiments. Furthermore, we established a C4‐2 cell line that can persistently overexpress NAT10 (lv‐NAT10) (**Figure** **2**A). Cell counting kit‐8 (CCK‐8) assays showed that proliferation was significantly reduced in NAT10‐knockdown cells but increased in cells overexpressing NAT10 (Figure 2B). Accordingly, the colony‐forming ability was significantly inhibited in the knockdown group of cells but significantly increased in the overexpression group of cells compared to control cells (Figure 2C).

The progression of the cell cycle is inextricably linked to the cell's ability to proliferate, so we subsequently used flow cytometry to detect changes in cell cycle distribution. The results showed that the knockdown of NAT10 in DU145 and PC‐3 cells induced G1 arrest, whereas ectopic expression of NAT10 in C4‐2 cells accelerated the cell cycle into the S phase (Figure 2D,E). After knocking down NAT10 in DU145 and PC‐3 cells, the expression of proteins p27 and p21, which inhibit cell cycle progression, significantly increased. In contrast, after the overexpression of NAT10 in C4‐2 cells, these proteins were significantly reduced (Figure 2F). These results indicate that NAT10 could promote cell cycle progression and subsequently enhance the proliferation of PCa cells by regulating cell cycle‐related proteins.

We also carried out experiments on apoptosis. The percentage of apoptosis in prostate cancer cells, however, was not found to be impacted by NAT10 (Figure S1A, Supporting Information).

### 2.3 NAT10 Facilitates PCa Cell Migration In Vitro

**Figure 3:**
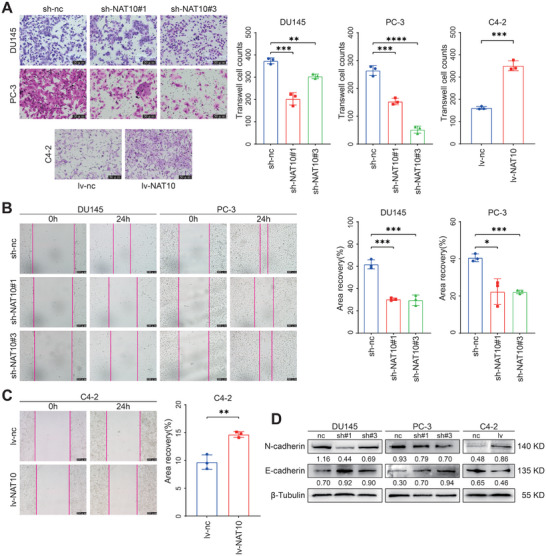
NAT10 facilitates PCa cell migration in vitro. A) Effect of knockdown or overexpression of NAT10 on the migration ability of PCa cells detected by transwell migration assay. Scale bar = 50 µm. B,C) Effect of knockdown or overexpression of NAT10 on the wound‐healing ability of PCa cells. Scale bar = 200 µm. D) Expression of EMT‐related proteins in PCa cell lines detected by the Western blot in PCa cell lines with or without knocking down or overexpressing NAT10.

To evaluate the ability of NAT10 in regulating PCa cell migration, transwell and wound‐healing assays were performed. The results of in vitro migration assays showed that knockdown of NAT10 inhibited cell migration of DU145 and PC‐3 cells, while overexpression of NAT10 promoted cell migration of C4‐2 cells (**Figure** **3**A). Wound‐healing experiments yielded the same results (Figure 3B,C). We postulated that NAT10 regulates the progression of EMT, which has been widely acknowledged as a crucial process in tumor migration. By using the Western blot, we evaluated the protein expression of EMT‐related markers. As expected, the knockdown of NAT10 resulted in a significant upregulation of the epithelial marker E‐cadherin and a significant downregulation of the mesenchymal marker N‐cadherin, suggesting that the progression of EMT was blocked. In contrast, overexpression of NAT10 showed the opposite effect (Figure 3D). These results suggest that NAT10 promotes PCa cell migration by regulating EMT.

### 2.4 Identification of NAT10‐Mediated Gene Alteration and N4‐Acetylation Modification Transcripts

**Figure 4:**
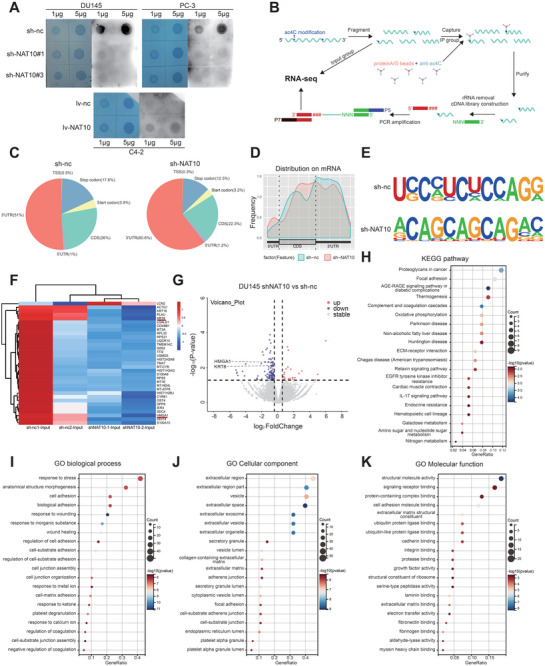
Identification of NAT10‐mediated gene alteration and N4‐acetylation modification transcripts. A) Effect of knockdown or overexpression of NAT10 on ac4C expression in PCa cells detected by dot blot. B) Schematic diagram of the flow of acRIP‐seq. C) Percentage distribution of ac4C peaks on RNA structures in control and NAT10 knockdown groups shown by pie charts. D) Metageneplot of the distribution of ac4C peaks on RNA structures in control and NAT10‐knockdown groups. E) Consensus motif of control and NAT10‐knockdown cells identified by hypergeometric optimization of motif enrichment (HOMER). F) Demonstration of the top 25% differential mRNA between sh‐nc and sh‐NAT10 group in DU145 shown by a heatmap. G) Volcano plot of mRNA expression changes between sh‐nc and sh‐NAT10 groups. H–K) Top 20 of KEGG pathway and GO enrichment analysis of differential genes in sh‐nc and sh‐NAT10 groups shown by bubble plots.

It has been shown that NAT10 could affect ac4C levels in Hela cells.^[^ ^9^ ^]^ Thus, we confirmed by dot blot experiments that knockdown of NAT10 also reduced ac4C levels in total RNA in PCa cell lines, while its overexpression did the opposite (**Figure** **4**A). Therefore, to investigate potential ac4C‐modified mRNA targets regulated by NAT10 in PCa, acetylated RNA immunoprecipitation sequence (acRIP‐seq) assays were performed on mRNAs isolated from the NAT10 stable knockdown cells (sh‐NAT10) and controls (sh‐nc) of the DU145 cell line (Figure 4B). The ac4C peaks in the cells of sh‐nc and sh‐NAT10 groups were dispersed in the 3′‐UTR (51.0% vs. 60.6%), CDS (26.0% vs. 22.3%), termination codon (3.9% vs. 3.2%), and 5′UTR (1.0% vs. 1.2%). Overall, the reduction of the ac4C peak in NAT10 knockdown cells was mainly located in the CDS region (Figure 4C,D). In line with earlier research, the most common ac4C sequence “CXXCXXCXX” was significantly enriched in ac4C peaks in both groups of cells (Figure 4E).

In addition, to elucidate the pathways and cellular functions of PCa cells that may be affected by NAT10, we analyzed the differential genes between sh‐nc and sh‐NAT10 in the input group (Figure 4F,G). Kyoto encyclopedia of genes and genomes (KEGG) pathway analysis and gene ontology (GO) analysis of differential genes demonstrated that knockdown of NAT10 can affect biological functions of PCa cells including focal adhesion, ECM‐receptor interaction, cell adhesion, cadherin binding, and others, which are critical for tumor progression (Figure 4H–K).

### 2.5 mRNA ac4C Modification Increases the Stability of Targets in PCa Cells

**Figure 5:**
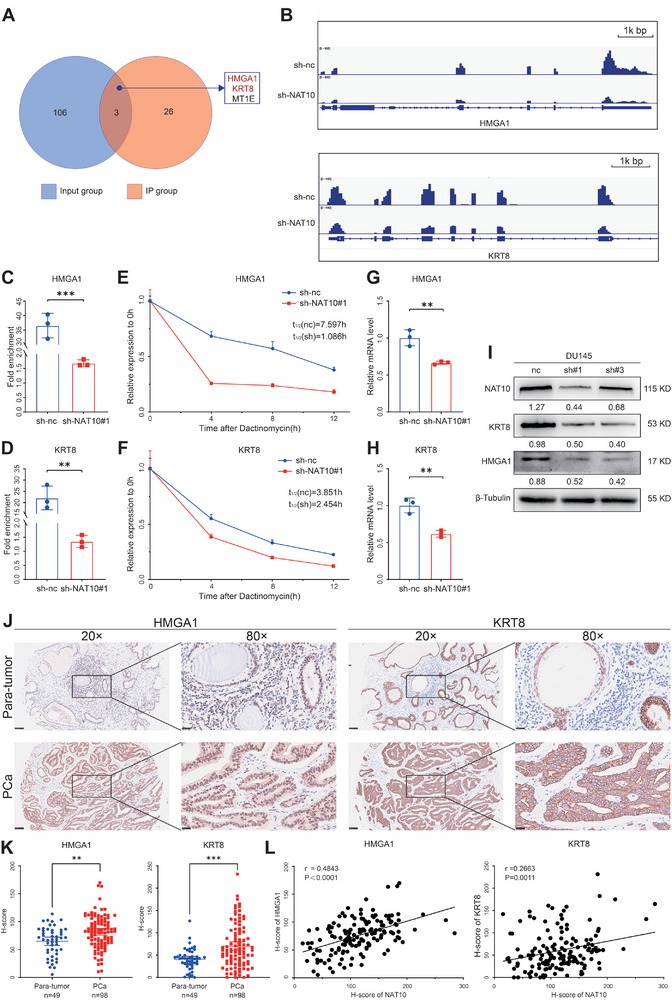
mRNA ac4C modification increases the stability of targets in PCa cells. A) Venn diagram showing the intersection of IP and input group differential genes in acRIP‐seq. B) Read distributions across target transcripts from acRIP‐seq are tracked by the IGV. C,D) Analyzing ac4C‐modified mRNA changes in HMGA1 and KRT8 with or without NAT10 knockdown by RIP‐qPCR. E,F) qRT‐PCR showed the effect of NAT10 knockdown on the stability of HMGA1 and KRT8 mRNA in DU145. G–I) Changes in abundance of HMGA1 and KRT8 in DU145 after knockdown of NAT10 detected by qRT‐PCR and the Western blot. J,K) Comparison of HMGA1 and KRT8 expression in para‐tumor and PCa samples in the TMA. L) Correlation analysis of HMGA1 or KRT8 and NAT10. Scale bar = 25 and 100 µm.

NAT10 can generate ac4C by acetylating mRNA, thereby affecting its stability and exerting its biological function. Since NAT10 positively mediates ac4C modification, only ac4C peaks with decreased abundance (termed ac4C hypo‐peaks) upon NAT10 knockdown were theoretically anticipated to include genuine targets of NAT10. We assessed whether these ac4C hypo‐peaks were associated with differentially expressed mRNA genes in the input analysis. After knocking down NAT10, we analyzed the genes that could encode proteins, and there were 109 genes with significantly downregulated expression levels of mRNAs in the input group, while there were 29 genes with significantly reduced ac4C modification sites in the immunoprecipitation (IP) group. The intersection allowed us to identify three potential targets (**Figure** **5**A). In addition, we found that the expression of HMGA1 and KRT8 was significantly higher in PCa tissues than in normal tissues by bioinformatics analysis, whereas metallothionein 1E (MT1E) was not (Figure S1B, Supporting Information). Through reviewing the literature and preliminary phenotyping studies, we identified HMGA1 and KRT8 as the subsequent research targets. From the peak maps of the sequencing results visualized by the Integrative Genomics Viewer (IGV), it was observed that after knocking down NAT10, the ac4C peaks of HMGA1 and KRT8 underwent a significant decrease (Figure 5B). To further validate our acRIP‐seq results, we performed acRIP‐qPCR assays and found that the ac4C abundance of HMGA1 and KRT8 was significantly reduced upon NAT10 knockdown in DU145 and PC‐3 cells (Figure 5C,D; Figure S1C, Supporting Information). Accordingly, overexpression of NAT10 increased the ac4C abundance of HMGA1 and KRT8 in C4‐2 cells (Figure S1D, Supporting Information). The mRNA half‐life assay showed that the stability of HMGA1 and KRT8 was significantly reduced by downregulating NAT10 (Figure 5E,F). We also detected the expression of HMGA1 and KRT8 by quantitative real‐time polymerase chain reaction (qRT‐PCR) and the Western blot. The results showed that after knocking down NAT10, the mRNA level and protein expression of HMGA1 and KRT8 were significantly decreased (Figure 5G–I). Immunohistochemistry (IHC) of TMA showed that the expression of HMGA1 and KRT8 was significantly higher in PCa than in paraneoplastic tissues (Figure 5J,K). Furthermore, there was a positive correlation observed between the expression of HMGA1 or KRT8 and NAT10 (Figure 5L). These data suggest that NAT10 can catalyze the ac4C modification of mRNA of HMGA1 and KRT8, thus improving their stability and expression.

### 2.6 HMGA1 and KRT8 are Functional Targets of NAT10 in PCa Cells

**Figure 6:**
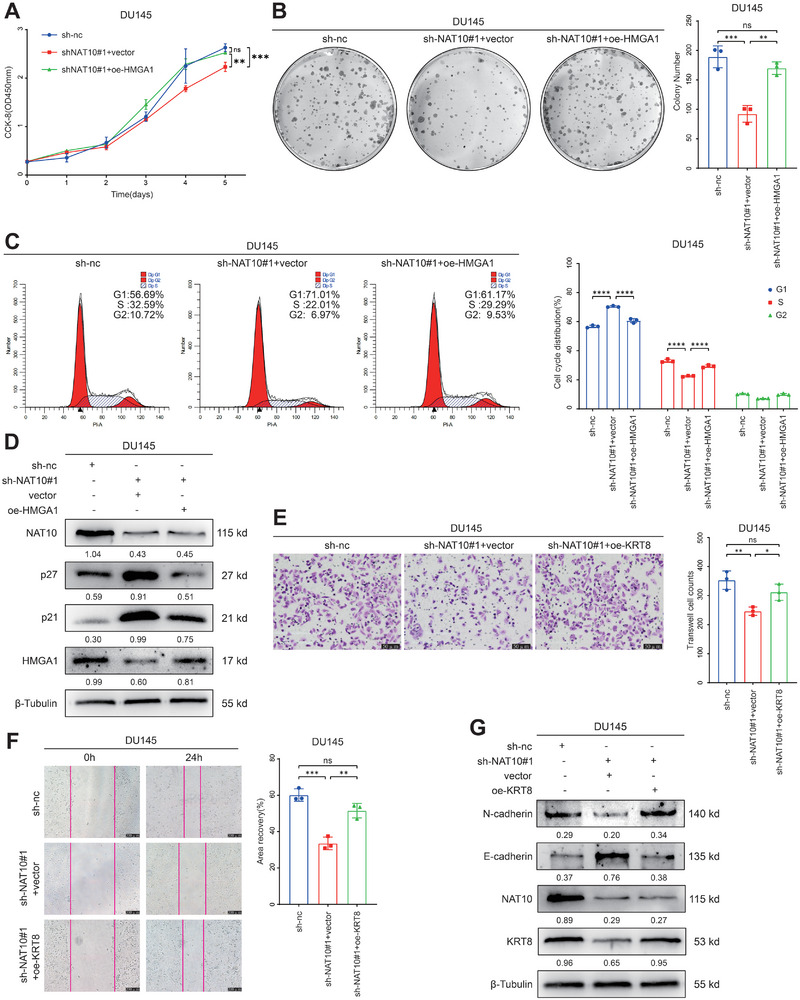
HMGA1 and KRT8 are functional targets of NAT10 in PCa cells. A–D) Effects of co‐transfection of sh‐NAT10 and oe‐HMGA1 on cell proliferation and cell cycle by CCK‐8 assay, colony formation assay, flow cytometry, and the Western blot. E–G) Effects of co‐transfection of sh‐NAT10 and oe‐KRT8 on cell migration and EMT by transwell migration assay, wound‐healing assay, and Western blot.

To verify the biological significance of HMGA1 and KRT8 in mediating NAT10 function in PCa, we performed rescue experiments with recombinant plasmids. The results of CCK‐8 and colony formation experiments showed that the proliferative capacity of shNAT10#1 DU145 cells was restored after delivery of the recombinant somatic plasmid containing HMGA1 to DU145 cells (**Figure** **6**A,B). Moreover, flow cytometry results showed that the G1/S phase‐blocking phenotype caused by the knockdown of NAT10 could be reverted by HMGA1 (Figure 6C). Western blot results also showed that after overexpression of HMGA1, the expression of key cell cycle proteins p27 and p21 all underwent significant reversion (Figure 6D). Additionally, following NAT10 inhibition, re‐expression of KRT8 reversed the effects of reduced cell migration ability (Figure 6E,F). Moreover, the expression of EMT‐related proteins, E‐cadherin and N‐cadherin, could be reverted (Figure 6G). Furthermore, rescue experiments with PC‐3 cells showed the same outcomes (Figure S2, Supporting Information). We designed and validated siRNA sequences targeting HMGA1 and KRT8, and conducted rescue experiments in C4‐2 cells overexpressing NAT10. Results demonstrate that knockdown of HMGA1 and KRT8 respectively reversed the promoting effects of NAT10 on proliferation and migration of C4‐2 cells (Figure S3, Supporting Information).

We also performed rescue experiments on the DU145 cell line to assess the migratory function of HMGA1. However, its rescue capability was not as pronounced compared to KRT8, which was able to completely reverse the changes in cell migration caused by NAT10 (Figure S4A, Supporting Information). As for the rescue experiments on KRT8 regarding proliferation function, based on our current results, it does not seem to mediate the changes in cell proliferation caused by NAT10 (Figure S4B, Supporting Information). Therefore, in our subsequent experiments, we primarily focused on the effect of HMGA1 on proliferative ability and the effect of KRT8 on migratory ability.

Taken together, these findings demonstrate that NAT10 regulates the biological functions of PCa cells through ac4C modification of target mRNAs.

### 2.7 Knockdown of NAT10 Inhibits PCa Proliferation and Metastasis In Vivo

**Figure 7:**
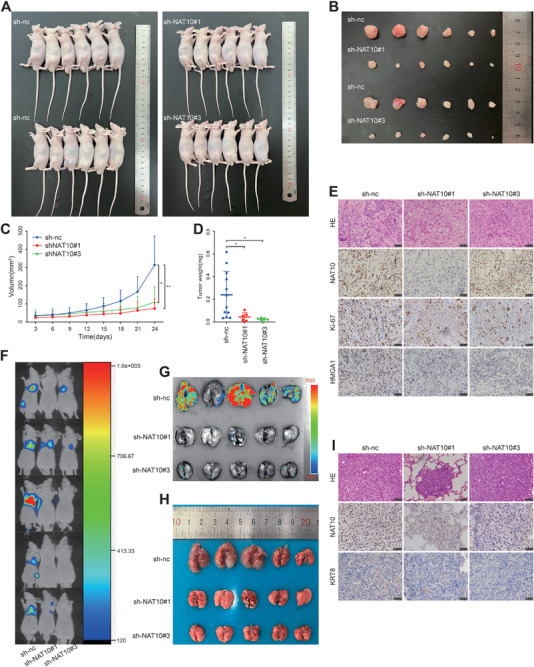
Knockdown of NAT10 inhibits PCa proliferation and metastasis in vivo. A) Gross view of the xenograft tumor in nude mice. B) Gross view of the xenograft tumors. C) Volume changes of the xenograft tumors. D) Final weights of the xenograft tumors. E) Typical images of xenograft tumors with HE staining and NAT10, Ki‐67 and HMGA1 IHC staining. Scale bar = 20 µm. F,G) The luciferase activities of lung metastasis were measured by an in vivo imaging system. H) Gross view of pulmonary metastasis. I) Typical images of pulmonary metastasis with HE staining and NAT10 and KRT8 IHC staining. Scale bar = 20 µm.

To investigate the role of NAT10 in regulating tumor proliferation and metastasis in vivo, we performed subcutaneous tumor xenograft in male BALB/c nude mice. We constructed NAT10‐knockdown DU145 cells that stably expressed luciferase and injected them subcutaneously into the mice (**Figure** **7**A). The results showed that the knockdown of NAT10 effectively inhibited the growth of prostate tumors, which was reflected by a significant reduction in the volume and weight of the tumors compared to the control group (Figure 7B–D). The xenografts were then dissected and isolated, and hematoxylin‐eosin (HE) staining experiments were applied to observe the histopathological features of the tumor tissues. Additionally, IHC staining was performed. It was found that the expression of Ki‐67 proliferation antigen and HMGA1 was significantly weaker in the NAT10 knockdown tumor tissues compared with the control group (Figure 7E).

We also constructed a tumor metastasis model by injecting NAT10‐knockdown DU145 cells intravenously into the mice to validate the effect of NAT10 on PCa metastasis. After 55 days of tumor cell injection, a reduction in lung metastatic sites was observed in both sh‐NAT10 groups compared to the sh‐nc group, which was reflected by the luciferase signal (Figure 7F). The lung organs were then dissected, and metastasis was further verified using an in vivo imaging system (Figure 7G,H). In addition, HE staining confirmed lung metastasis. As shown by IHC staining, KRT8 expression was significantly weaker in NAT10‐knockdown lung metastatic tissues compared to controls (Figure 7I). These experiments suggest that mice treated with sh‐NAT10 cells produce less lung colonization than sh‐nc cells.

Overall, consistent with the phenotype found in vitro, the target genes HMGA1 and KRT8 were inhibited by knocking down NAT10 in the mouse model, thereby inhibiting PCa proliferation and metastasis in vivo.

## 3 Discussion

In this study, we found that NAT10 is highly expressed in PCa and could serve as a predictor of the occurrence and poor prognosis of PCa. We carried out loss‐of‐function and gain‐of‐function experiments to clarify the crucial role that NAT10 plays in PCa. Our findings demonstrated that shRNA‐mediated silencing of NAT10 expression in PCa cells significantly decreased cell proliferation and migration, and inhibited entry into the S phase of the cell cycle. On the contrary, ectopic expression of NAT10 augmented the aggressive behavior of PCa cells. In in vivo studies using murine models, NAT10 knockdown resulted in inhibition of tumorigenesis and lung metastasis. These observations emphasize the critical role of NAT10 in PCa proliferation and metastasis.

Although it has been demonstrated that NAT10 is associated with the regulation of DNA replication in PCa,^[^ ^23^ ^]^ the present study has further refined the function of NAT10 in PCa from the perspective of post‐transcriptional modification. We also demonstrated the feasibility of using NAT10 as a diagnostic indicator of PCa by IHC at the tissue protein level.

Our study found that the knockdown of NAT10 resulted in a significant G1/S‐phase block in PCa cells, and the G1/S‐phase checkpoint is critical for cell proliferation.^[^ ^24^, ^25^, ^26^ ^]^ It has been shown that p53, as a transcription factor, activates downstream p21 transcription and inhibits several cell cycle protein‐dependent kinases, thereby blocking the progression of the G1/S phase of the cell cycle.^[^ ^27^, ^28^, ^29^ ^]^ In contrast, p27, a member of the family of cyclin‐dependent kinase proteins, similar to p21, binds to several cyclin complexes, thereby inhibiting their function and reinforcing the G1/S checkpoint of the cell cycle.^[^ ^30^, ^31^, ^32^, ^33^ ^]^ In consistent with these previous reports, the protein expression of p27 and p21 were significantly upregulated after knocking down NAT10. The results of flow cytometry also showed that NAT10 could promote the cell cycle progression of PCa cells.

Tumor cell migration is an important feature of malignancy, and has been shown to be associated with the EMT process in cancer cells in a variety of cancers.^[^ ^34^, ^35^, ^36^, ^37^ ^]^ EMT was first proposed in the 1980s to be indispensable during embryonic development, and its process is reactivated during wound healing, fibrosis, and cancer progression.^[^ ^38^ ^]^ EMT is a complex molecular and cellular program in which epithelial cells lose their differentiation features, such as cell‐cell adhesion, planar and apical‐basal polarity, and acquire mesenchymal features, such as motility, invasiveness, and resistance to apoptosis.^[^ ^39^ ^]^ The key protein molecules that we use to determine whether cells undergo EMT are the loss of the epithelial marker E‐cadherin and the acquisition of the mesenchymal marker N‐cadherin at the molecular level.^[^ ^40^ ^]^ In our study, we found that NAT10 could significantly improve the migration ability of PCa cells through EMT. We explored the effect of NAT10 on the EMT process of PCa cells through Western blot experiments. The results showed that the EMT process of cells was blocked after knocking down NAT10, and the opposite was true after overexpressing NAT10, suggesting that NAT10 can significantly enhance the migration ability of PCa cells by promoting their EMT process.

In the process of mechanism exploration, we screened mRNAs whose abundance as well as ac4C modification were decreased after the knockdown of NAT10 based on the results of acRIP‐seq. By taking the intersections of gene analysis, we obtained three genes, HMGA1, KRT8, and MT1E. By searching the literature, we found that HMGA1 was a structural protein of chromatin that is nonhistone and lacks transcriptional activity. By changing the DNA structure, it primarily serves as a regulator. Numerous investigations have verified that HMGA1 controls genes linked to tumors in the digestive, urinary, reproductive, and hematopoietic systems. Under external stimulation, it will produce effects through the pathways of Wnt/β‐catenin, phosphatidylinositol 3 kinase/protein kinase B (PI3K/Akt), Hippo, and MAP kinase‐ERK kinase/extracellular‐signal‐regulated kinase (MEK/ERK). Furthermore, HMGA1 influences cancer cell aging, apoptosis, autophagy, and resistance to chemotherapy, all of which are connected to carcinogenesis.^[^ ^41^ ^]^ Especially, it was found to target the cell cycle and exert its pro‐cancer function in other cancers.^[^ ^42^, ^43^ ^]^ KRT8 is mostly expressed in the basal side of epithelial cells and is a prominent component of the intermediate filament cytoskeleton.^[^ ^44^ ^]^ Recently, it has been discovered that KRT8 expression abnormalities are linked to tumor progressions, including drug resistance, cell migration, and cell adhesion.^[^ ^45^, ^46^, ^47^ ^]^ In particular, KRT8 can promote cancer progression by facilitating the EMT process.^[^ ^48^, ^49^ ^]^ However, in PCa studies, downregulation of MT1E is a potential feature of aggressive PCa.^[^ ^50^ ^]^ Therefore, we chose HMGA1 and KRT8 as the targets of our subsequent study. The peak plot analysis of acRIP‐seq and the results of RNA immunoprecipitation (RIP) experiments showed that after knocking down NAT10, the ac4C modifications of HMGA1 and KRT8 were both significantly decreased in cells. Their mRNA stability was significantly downregulated, resulting in decreased mRNA and protein abundance, which corroborated with previous studies.^[^ ^9^ ^]^ After overexpression of HMGA1 and KRT8, the altered proliferation and migration functions of PCa cells caused by NAT10 knockdown regressed. Therefore, HMGA1 and KRT8 are downstream targets of NAT10 for it to exert mRNA acetylation modification function and affect PCa function.

**Figure 8:**
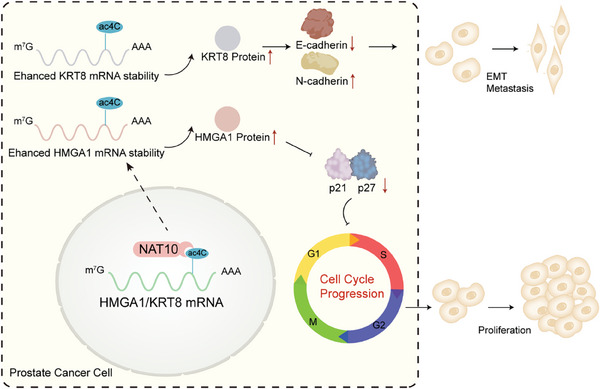
The schematic model for the roles of NAT10 in PCa.

In conclusion, our research showed that NAT10 is critical for PCa carcinogenesis and metastasis. NAT10 could enhance the stability of target genes by catalyzing ac4C modification of their mRNAs. NAT10 could mediate the proliferation of PCa cells through HMGA1‐mediated changes in the cell cycle and regulate the migration ability of PCa through KRT8‐mediated changes in EMT (**Figure** **8**). Additionally, NAT10 could be used as a molecular marker to identify PCa cell proliferation and migration.

## 4 Experimental Section

### Bioinformatics Analysis

All bioinformatics analyses were run through the R (version 4.2.1) software. Data on PCa tissues that are accessible to the public was obtained from TCGA. At the median of expression values, NAT10 expression was stratified into two groups: high and low. The Kaplan‐Meier method was utilized to estimate the disease specific survival (DSS) and disease free survival (DFS) of patients. The proportional risk hypothesis was tested, and survival regression was fitted using the survival package. The survminer and ggplot2 packages were used to visualize the results. The GO annotations for the genes in the R package org.Hs.eg.db (version 3.1.0) and the most recent gene annotations for the KEGG Pathway was used, which it was obtained using the KEGG rest API (https://www.kegg.jp/kegg/rest/keggapi.html). Gene set enrichment results were obtained via enrichment analysis utilizing the R package clusterProfiler (version 3.14.3).

### Clinical Samples and IHC

IHC for NAT10 was performed on HProA150CS01 human PCa TMA obtained from Shanghai Outdo Biotech Company (Shanghai, China). The TMA contained 100 PCa samples and 50 adjacent normal tissues. The pathology grade, Gleason grade, Gleason score, TNM, and clinical stage for each case of the specimen were available. All clinicopathological data of enrolled patients were summarized in Tables S1 and S2 (Supporting Information). The procedure for IHC staining followed the previous publication.^[^ ^51^ ^]^ Briefly, NAT10 (1:250, ab194297, Abcam, Cambridge, UK), HMGA1 (1:500, ab129153, Abcam), KRT8 (1:500, ab53280, Abcam), or Ki‐67 (1:500, ab15580, Abcam) were incubated with TMA or tumor tissue slices for at least 16 h at 4 °C. The cell number and staining intensity of each sample in the TMA were quantitatively analyzed by QuPath software (https://qupath.github.io). The staining intensity of NAT10 was categorized into four grades (− to +++) according to set thresholds, from which the H‐score of each sample was calculated, and its correlation with clinicopathological parameters was assessed.

### Cell Line

Five PCa cell lines (PC‐3, DU145, C4‐2, 22Rv1, and LNCaP) as well as the human immortalized prostate epithelial cell line RWPE‐1 were bought from the Chinese Academy of Sciences' Stem Cell Bank. Short tandem repeat profiling was used to identify each cell line. These cancer cells were cultured in RPMI 1640 medium with 10% fetal bovine serum (No. 10270‐106, Gibco, New York, United States). RWPE‐1 was grown in keratinocyte serum‐free medium (No. 10744‐019, Gibco) with 5 ng mL^−1^ epidermal growth factor (No. 10450‐013, Gibco). All cell lines were kept in 5% CO2 at 37 °C.

### Plasmid Construction and Lentiviral Infection

Following the manufacturer's instructions, stable cell lines expressing shRNA‐NAT10 and NAT10 were produced using lentiviral vectors from Qingke Biotech (Shanghai, China) and Hambio (Shanghai, China), respectively. Puromycin (2 µg ml^−1^) was used to select the lentivirus‐transfected cells for one week. In addition, plasmids that overexpress HMGA1 (oe‐HMGA1) or KRT8 (oe‐KRT8) were synthesized by Hambio. All the target sequences were shown in Table S3 (Supporting Information).

### Western Blot

According to the manufacturer's (KGB5303‐100, KeyGEN BioTech, Jiangsu, China) methodology, proteins were isolated from cells. The protein concentrations in the cell lysates were measured using the BCA Protein Assay Kit (Beyotime Biotechnology). Western blot was carried out as previously stated.^[^ ^52^ ^]^ In addition to the antibodies used in IHC, the antibodies used in this study included anti‐p27 Kip1 (#3686, Cell Signaling, Massachusetts, United States), anti‐p21 Waf1/Cip1 (#2947), anti‐E‐cadherin (#3195) and anti‐N‐cadherin (#13 116).

### RNA Extraction and qRT‐PCR Assays

The main experimental steps were the same as in a previous study.^[^ ^52^ ^]^ The primers used in the study were listed in Table S4 (Supporting Information).

### Cell Proliferation

CCK‐8 (FD3788, Fude, Hangzhou, China) was used to measure cell viability in accordance with the manufacturer's recommendations. Cells were planted onto 96‐well plates at a density of 2×10^3^ cells per well. Every 24 h thereafter, 10% v/v CCK‐8 was added to each plate and the cells were incubated at 37 °C for 2 h. A microplate reader (EXL800, BioTek Instruments, Vermont, United States) was then used to measure the optical density (OD) at 450 nm. Each experiment was repeated in at least 3 wells per time point.

### Colony Formation

800 PCa cells were seeded into each well in a 6‐well plate and placed in an incubator for two weeks. At the end of the incubation, the cells were fixed with 4% paraformaldehyde and stained with Giemsa.

### Cell‐Cycle Analysis

Cell‐cycle analysis was executed as described previously.^[^ ^53^ ^]^ In a nutshell, PCa cells were fixed with ice‐cold 70% ethanol at 4 °C overnight after being digested with 0.25% trypsin/ethylene‐diamine‐tetraacetie‐acid (EDTA) solution. Cells were then stained with 50 µg mL^−1^ propidium iodide (PI) (KGA9101‐100, KeyGEN BioTech). Flow cytometry (FACS Calibur, Becton Dickinson, New Jersey, United States) was used to measure the deoxyribonucleic acid (DNA) content of the cells in each group.

### Transwell Migration Assay

Transwell inserts (Costar, Corning, Cambridge, MA, United States) with a chamber (8.0 µm pore size) were used for the cell migration assay. 500 µl of complete medium was added to the lower chamber of the insert. 300 µl of serum‐free medium containing 5×10^4^ cells were added to the upper chamber. After being placed in an incubator for an appropriate amount of time (DU145 12 h, PC‐3 48 h, C4‐2 48 h), the cells were fixed with 4% paraformaldehyde and stained with Giemsa. Subsequently, the cells on the upper chamber surface of the membrane were removed and photographed with an inverted microscope. At least 3 fields of view were randomly selected for photographing and counted with Image J software.

### Wound Healing Assay

PCa cells were seeded into a 6‐well plate until confluence reached 90%. Linear wounds were made with a 100 µl plastic pipette tip. The cell debris was washed away with phosphate buffer saline (PBS) and serum‐free medium was added. At 0 and 24 h, wounds were observed and photographed at the same location using an inverted microscope (Olympus IX71, Jingtong, Suzhou, China). At least 3 positions were recorded in each group. The wound area was measured using Image J software and the percentage of area recovery was calculated.

### Mice xenograft and Tumor Metastasis

All animal experiments employed BALB/c male mice (4 weeks) raised under specific pathogen‐free conditions, and all experimental animal surgeries were authorized by the Southern Hospital's Animal Ethics Committee (IACUC‐LAC‐20230519‐003). The right or left dorsal aspect of the back legs of mice were implanted with 5×10^6^ DU145 NAT10 stable knockdown cells or controls to create the subcutaneous xenograft model. Tumor size was measured every 3 days, and the volume was calculated using the following formula: volume = 0.52×length×width^2^. To create a metastatic model, 2×10^6^ cells that had been transfected with sh‐NAT10 and sh‐nc were resuspended in 100 µl of PBS and injected into the mouse tail vein. After eight weeks, the mice were put under anesthesia and given an intraperitoneal injection of D‐luciferin (B6040, Apexbio, Texas, United States). The luciferase signal was then visible using the in vivo imaging system (Caliper Life Sciences, Massachusetts, United States). To further identify the characteristics of xenograft tumors and lung metastasis, IHC and HE staining were used.

### Dot Blot

A drop of diluted RNA was added to the Hybond‐N+ membrane (GE Health, Illinois, United States) and placed under UV light to crosslink the RNA to the membrane. The membrane was incubated with an anti‐ac4C antibody (ab252215, Abcam), which was subsequently warmed with horseradish peroxidase‐conjugated secondary antibody, and the signal from the blotting was visualized using the enhanced chemiluminescence Western Blotting Detection Kit (Thermo Fisher Scientific). Finally, the membrane was treated with methylene blue staining solution as a loading control.

### acRIP‐Seq

To detect the level of ac4C modification in all genes, acRIP‐seq was performed. This technology was provided by Guangzhou Epibiotek Co., Ltd. (Guangzhou, China). The main process was to interrupt the RNA of the cells at a high temperature and incubate it with an anti‐ac4C antibody conjugated with magnetic beads. After purification and removal of rRNA, the RNA was sequenced. Ac4C peaks were identified using exomePeak R package (v2.13.2) under parameters: “PEAK CUTOFF PVALUE = 0.05, PEAK_ CUTOFF_ FDR = NA, FRAGMENT_LENGTH = 200”.

### RIP

All experimental steps were performed according to the instructions of the Magna RIP Kit (MilliporeSigma, Massachusetts, United States). Briefly, the cell sediment was mixed with RIP lysate. 50 µl of the supernatant was taken as the input group, while 850 µl of the supernatant was incubated with magnetic beads conjugated with either anti‐ac4C antibody or immunoglobin G (IgG) overnight at 4 °C. After the magnetic beads were washed with RIP buffer, the RNA was eluted off at 55 °C with proteinase K. The RNA was then extracted with phenol‐chloroform‐isoamyl alcohol. Finally, the RNA was analyzed for relative quantification by qRT‐PCR.

### mRNA Stability Assay

The sh‐NAT10 and sh‐nc cell lines were treated with the transcription inhibitor actinomycin D (2 µg ml^−1^) at indicated times before cell collection. Trizol was used to capture total RNA and qRT‐PCR to quantify the relative levels of target mRNAs.

### Statistical Analysis

All data were presented as mean ± SD unless otherwise specified. For the statistical analysis in this work, GraphPad Prism9.1.0 (GraphPad Software, California, United States) was used. For the data analysis between two groups whose data follow a normal distribution, the two‐tailed Student's t‐test was used. The Mann‐Whitney test was used for data that did not follow normal distribution. The Kaplan‐Meier survival curve was calculated using the log‐rank test. Groups of continuous variables were analyzed using a one‐way analysis of variance. Pearson's chi squared test and Yates's correction for continuity were used for categorical variable. Statistical significance was defined as a *p*‐value of < 0.05. *p < 0.05, **p < 0.01, ***p < 0.001, ****p < 0.0001. NS, no significant.

## Conflict of Interest

The authors declare no conflict of interest.

## Supporting information

Supporting Information

## Data Availability

The data that support the findings of this study are available from the corresponding author upon reasonable request.
