# Supplementary material for: NAT10 Promotes Prostate Cancer Growth and Metastasis by Acetylating mRNAs of HMGA1 and KRT8
Source: Adv Sci (Weinh). 2024 Jun 23;11(32):2310131. doi: 10.1002/advs.202310131 (PMC11348116; doi:10.1002/advs.202310131)
Supplement: Supplementary file 1 — Supporting Information [file ADVS-11-2310131-s001.docx]

Supporting Information

NAT10 promotes prostate cancer growth and metastasis by acetylating mRNAs of HMGA1 and KRT8

Kang-Jing Li, Yaying Hong, Yu-Zhong Yu, Zhiyue Xie, Dao-Jun Lv, Chong Wang, Tao Xie, Hong Chen, Zhe-Sheng Chen,* Jianwen Zeng,* and Shan-Chao Zhao*

Supplementary figures


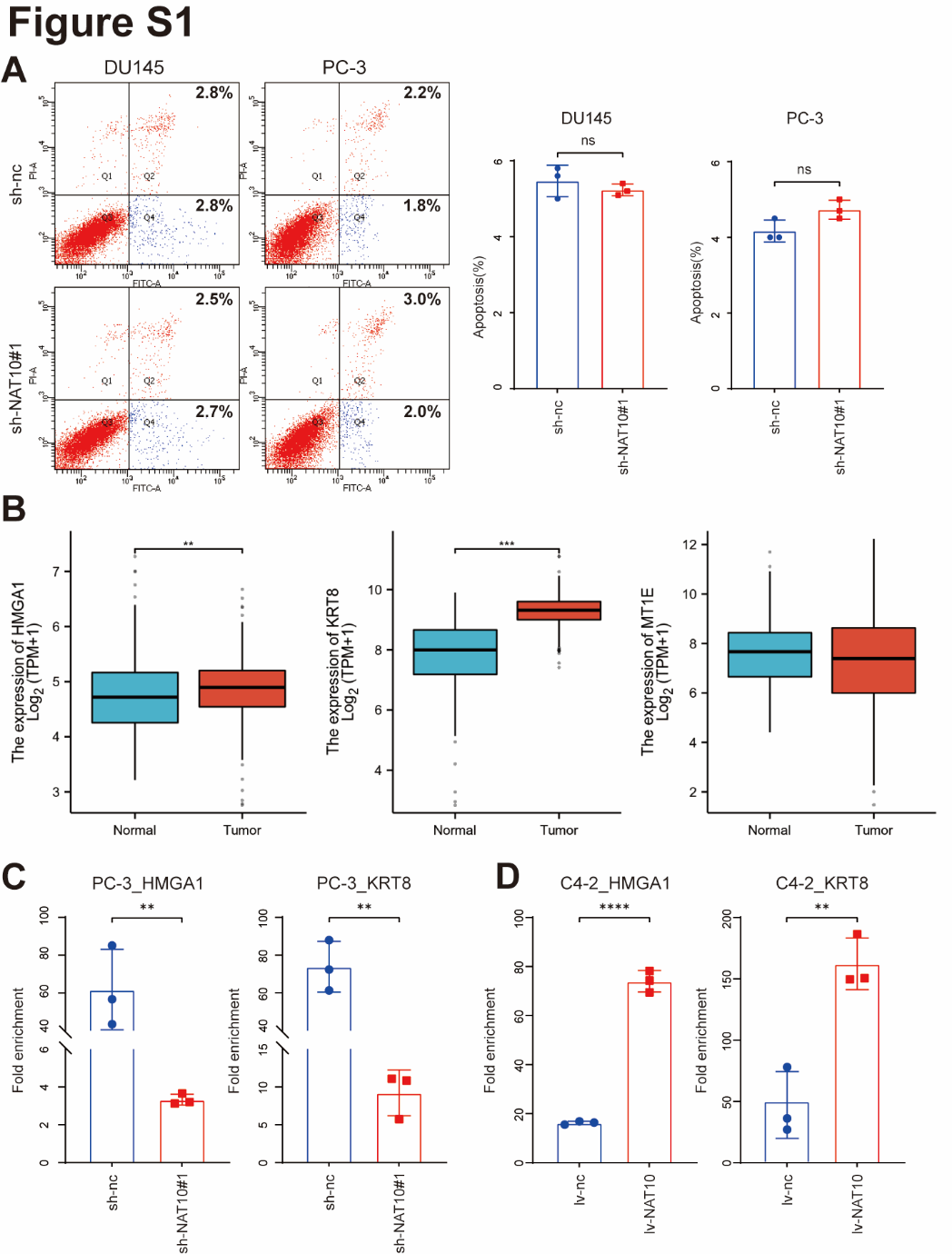


**Figure S1.** A) Effect of knockdown of NAT10 on apoptosis in prostate cancer cell lines. B) Expression of HMGA1, KRT8 and MT1E in the TCGA PCa database. C-D) acRIP-qPCR experiments for the C4-2 and PC-3 cell lines.


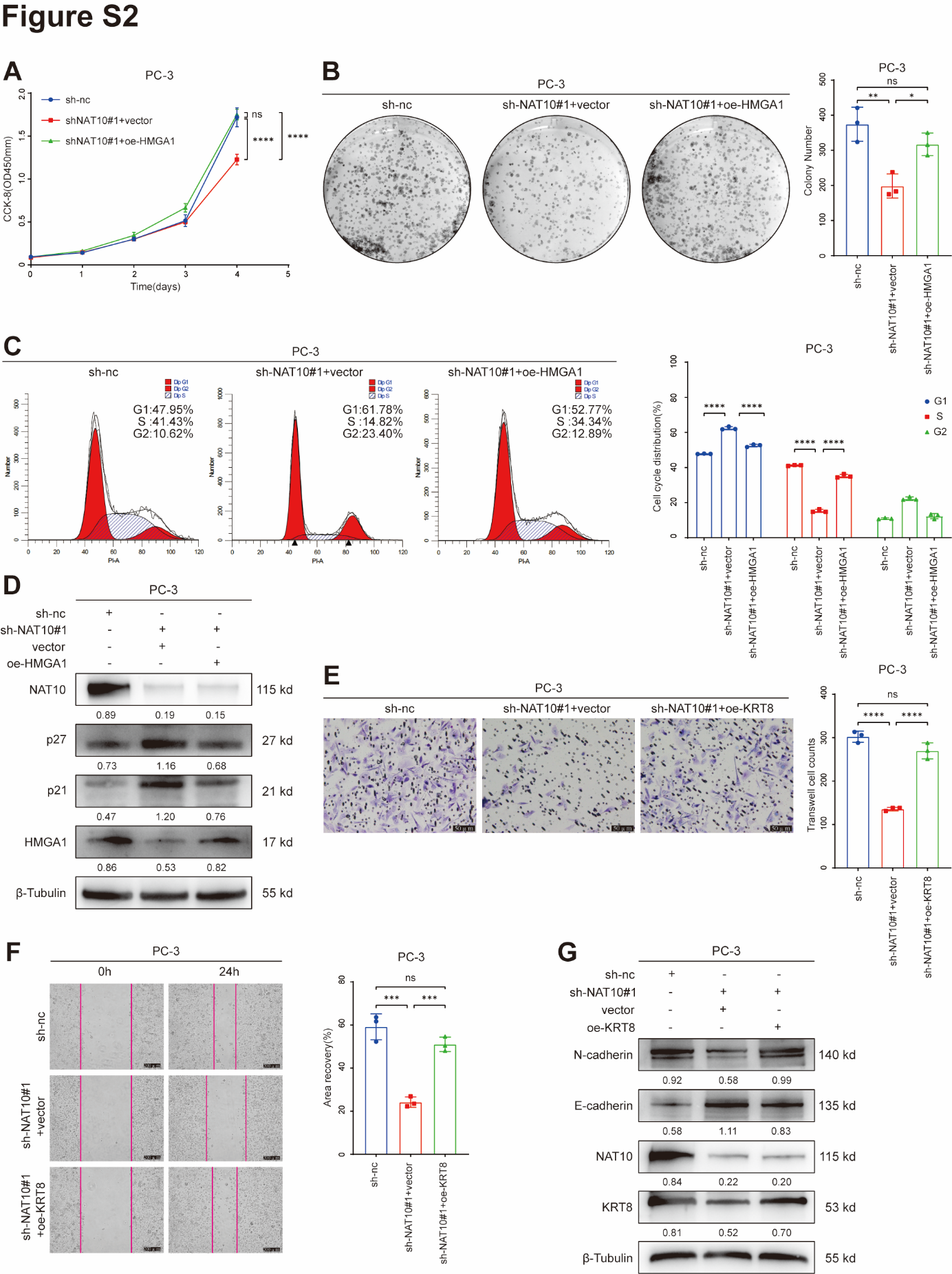


**Figure S2.** Rescue experiments with the PC-3 cell line.


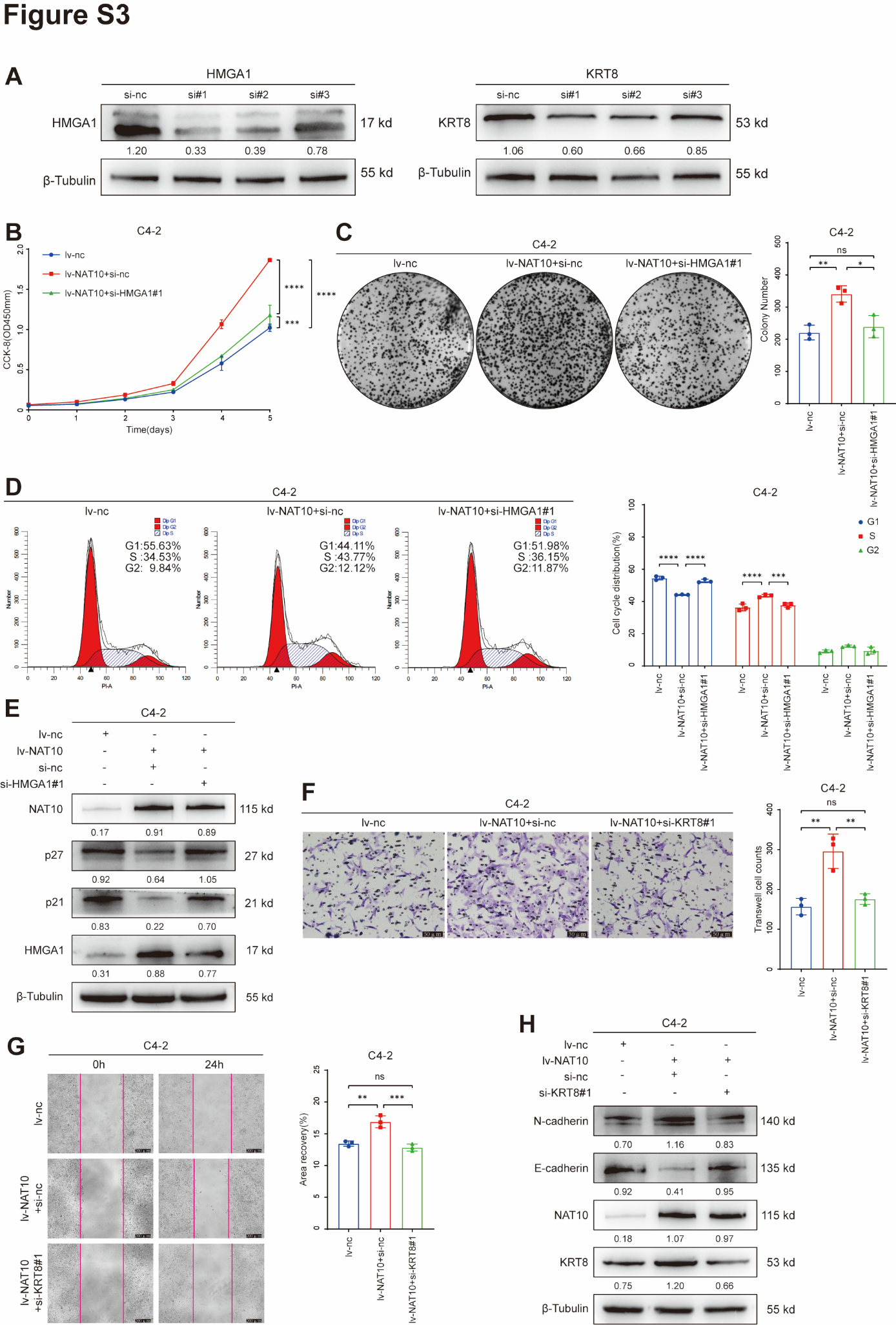


**Figure S3.** Rescue experiments with the C4-2 cell line.


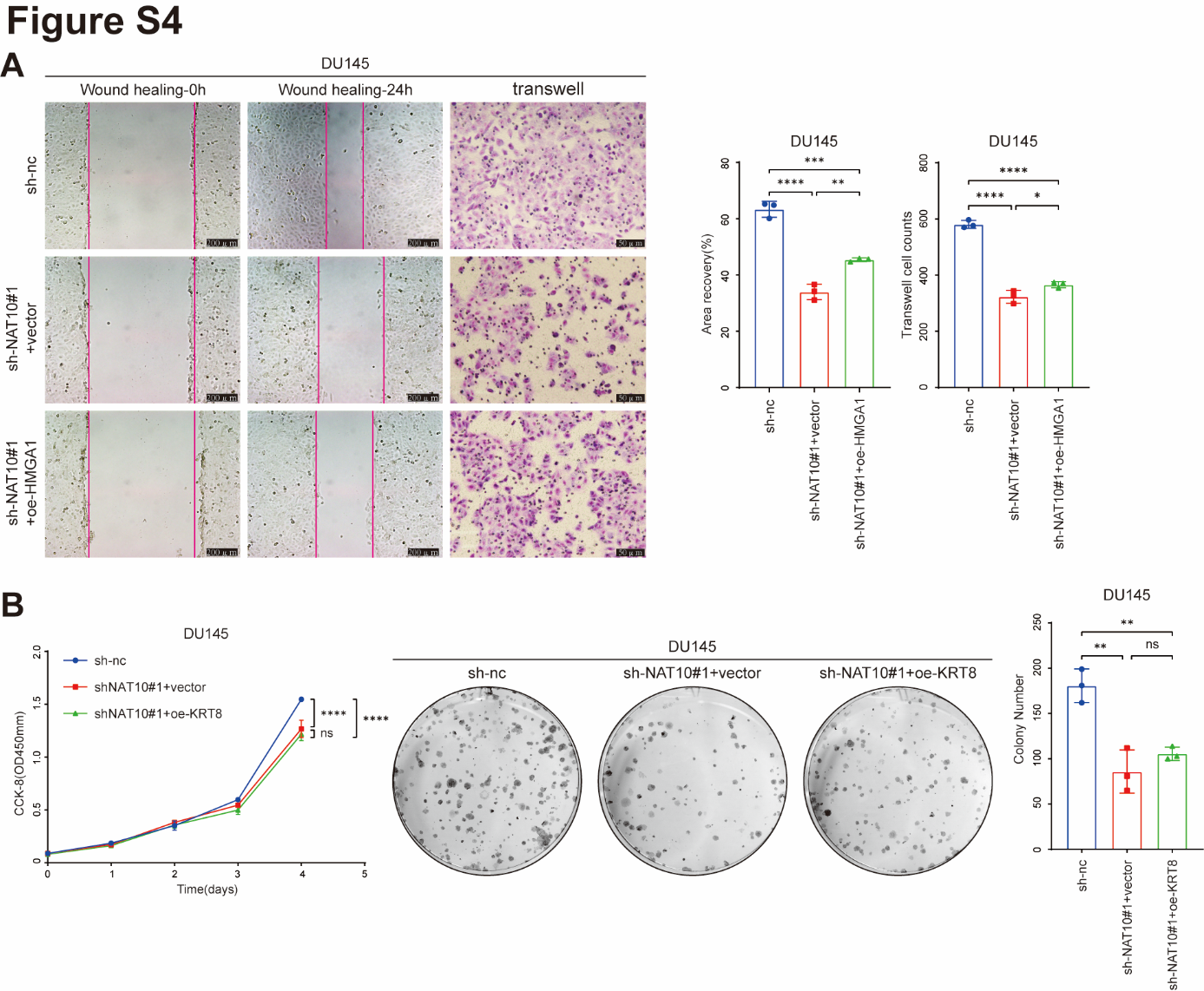


**Figure S4.** A) Effects of co-transfection of sh-NAT10 and oe-HMGA1 on cell migration by transwell migration assay and wound-healing assay. B). Effects of co-transfection of sh-NAT10 and oe-KRT8 on cell proliferation by CCK-8 assay and colony formation assay.

Supplementary tables

**Table S1. The information of patients for TMA**

| Sites | Specimens type | Age | gender | Clinical stage | Gleason Score | T | N | M |
| --- | --- | --- | --- | --- | --- | --- | --- | --- |
| A01 | Adenocarcinoma | 74 | **M** | **ⅡB** | 3+4=7 | **T2** | **N0** | **M0** |
| A02 | Adjacent normal prostate tissue | 74 | **M** | - | - | - | - | - |
| A03 | Adenocarcinoma | 70 | **M** | **ⅡB** | 3+4=7 | **T2** | **N0** | **M0** |
| A04 | Adjacent normal prostate tissue | 70 | **M** | - | - | - | - | - |
| A05 | Adenocarcinoma | 65 | **M** | **ⅡB** | 3+4=7 | **T2** | **N0** | **M0** |
| A06 | Adjacent normal prostate tissue | 65 | **M** | - | - | - | - | - |
| A07 | Adenocarcinoma | 76 | **M** | **ⅡB** | 3+4=7 | **T2** | **N0** | **M0** |
| A08 | Adjacent normal prostate tissue | 76 | **M** | - | - | - | - | - |
| A09 | Adenocarcinoma | 65 | **M** | **ⅡB** | 3+4=7 | **T2** | **N0** | **M0** |
| A10 | Adjacent normal prostate tissue | 65 | **M** | - | - | - | - | - |
| A11 | Adenocarcinoma | 73 | **M** | **ⅡB** | 3+4=7 | **T2** | **N0** | **M0** |
| A12 | Adjacent normal prostate tissue | 73 | **M** | - | - | - | - | - |
| A13 | Adenocarcinoma | 57 | **M** | **ⅡB** | 3+4=7 | **T2** | **N0** | **M0** |
| A14 | Adjacent normal prostate tissue | 57 | **M** | - | - | - | - | - |
| A15 | Adenocarcinoma | 73 | **M** | **ⅡC** | 4+3=7 | **T2** | **N0** | **M0** |
| A16 | Adjacent normal prostate tissue | 73 | **M** | - | - | - | - | - |
| B01 | Adenocarcinoma | 77 | **M** | **ⅡC** | 4+3=7 | **T2** | **N0** | **M0** |
| B02 | Adjacent normal prostate tissue | 77 | **M** | - | - | - | - | - |
| B03 | Adenocarcinoma | 68 | **M** | **ⅢA** | 3+3=6 | **T2** | **N0** | **M0** |
| B04 | Adjacent normal prostate tissue | 68 | **M** | - | - | - | - | - |
| B05 | Adenocarcinoma | 76 | **M** | **ⅢA** | 4+3=7 | **T2** | **N0** | **M0** |
| B06 | Adjacent normal prostate tissue | 76 | **M** | - | - | - | - | - |
| B07 | Adenocarcinoma | 79 | **M** | **ⅢB** | 3+3=6 | **T4** | **N0** | **M0** |
| B08 | Adjacent normal prostate tissue | 79 | **M** | - | - | - | - | - |
| B09 | Adenocarcinoma | 78 | **M** | **ⅢB** | 3+3=6 | **T4** | **N0** | **M0** |
| B10 | Adjacent normal prostate tissue | 78 | **M** | - | - | - | - | - |
| B11 | Adenocarcinoma | 83 | **M** | **ⅢB** | 3+4=7 | **T3** | **N0** | **M0** |
| B12 | Adjacent normal prostate tissue | 83 | **M** | - | - | - | - | - |
| B13 | Adenocarcinoma | 81 | **M** | **ⅢB** | 3+4=7 | **T3** | **N0** | **M0** |
| B14 | Adjacent normal prostate tissue | 81 | **M** | - | - | - | - | - |
| B15 | Adenocarcinoma | 62 | **M** | **ⅢB** | 3+4=7 | **T3** | **N0** | **M0** |
| B16 | Adjacent normal prostate tissue | 62 | **M** | - | - | - | - | - |
| C01 | Adenocarcinoma | 76 | **M** | **ⅢB** | 3+4=7 | **T4** | **N0** | **M0** |
| C02 | Adjacent normal prostate tissue | 76 | **M** | - | - | - | - | - |
| C03 | Adenocarcinoma | 70 | **M** | **ⅢB** | 3+4=7 | **T3** | **N0** | **M0** |
| C04 | Adjacent normal prostate tissue | 70 | **M** | - | - | - | - | - |
| C05 | Adenocarcinoma | 63 | **M** | **ⅢB** | 3+4=7 | **T3** | **N0** | **M0** |
| C06 | Adjacent normal prostate tissue | 63 | **M** | - | - | - | - | - |
| C07 | Adenocarcinoma | 72 | **M** | **ⅢB** | 3+4=7 | **T3** | **N0** | **M0** |
| C08 | Adjacent normal prostate tissue | 72 | **M** | - | - | - | - | - |
| C09 | Adenocarcinoma | 68 | **M** | **ⅢB** | 3+4=7 | **T4** | **N0** | **M0** |
| C10 | Adjacent normal prostate tissue | 68 | **M** | - | - | - | - | - |
| C11 | Adenocarcinoma | 68 | **M** | **ⅢB** | 3+4=7 | **T3** | **N0** | **M0** |
| C12 | Adjacent normal prostate tissue | 68 | **M** | - | - | - | - | - |
| C13 | Adenocarcinoma | 69 | **M** | **ⅢB** | 4+3=7 | **T3** | **N0** | **M0** |
| C14 | Adjacent normal prostate tissue | 69 | **M** | - | - | - | - | - |
| C15 | Adenocarcinoma | 71 | **M** | **ⅢB** | 4+3=7 | **T4** | **N0** | **M0** |
| C16 | Adjacent normal prostate tissue | 71 | **M** | - | - | - | - | - |
| D01 | Adenocarcinoma | 72 | **M** | **ⅢB** | 4+3=7 | **T4** | **N0** | **M0** |
| D02 | Adjacent normal prostate tissue | 72 | **M** | - | - | - | - | - |
| D03 | Adenocarcinoma | 71 | **M** | **ⅢB** | 4+3=7 | **T3** | **N0** | **M0** |
| D04 | Adjacent normal prostate tissue | 71 | **M** | - | - | - | - | - |
| D05 | Adenocarcinoma | 71 | **M** | **ⅢB** | 4+3=7 | **T4** | **N0** | **M0** |
| D06 | Adjacent normal prostate tissue | 71 | **M** | - | - | - | - | - |
| D07 | Adenocarcinoma | 74 | **M** | **ⅢB** | 4+3=7 | **T3** | **N0** | **M0** |
| D08 | Adjacent normal prostate tissue | 74 | **M** | - | - | - | - | - |
| D09 | Adenocarcinoma | 64 | **M** | **ⅢB** | 4+3=7 | **T3** | **N0** | **M0** |
| D10 | Adjacent normal prostate tissue | 64 | **M** | - | - | - | - | - |
| D11 | Adenocarcinoma | 70 | **M** | **ⅢB** | 4+3=7 | **T3** | **N0** | **M0** |
| D12 | Adjacent normal prostate tissue | 70 | **M** | - | - | - | - | - |
| D13 | Adenocarcinoma | 72 | **M** | **ⅢB** | 3+5=8 | **T3** | **N0** | **M0** |
| D14 | Adjacent normal prostate tissue | 72 | **M** | - | - | - | - | - |
| D15 | Adenocarcinoma | 72 | **M** | **ⅢB** | 4+4=8 | **T4** | **N0** | **M0** |
| D16 | Adjacent normal prostate tissue | 72 | **M** | - | - | - | - | - |
| E01 | Adenocarcinoma | 75 | **M** | **ⅢB** | 4+4=8 | **T3** | **N0** | **M0** |
| E02 | Adjacent normal prostate tissue | 75 | **M** | - | - | - | - | - |
| E03 | Adenocarcinoma | 70 | **M** | **ⅢB** | 5+3=8 | **T3** | **N0** | **M0** |
| E04 | Adjacent normal prostate tissue | 70 | **M** | - | - | - | - | - |
| E05 | Adenocarcinoma | 72 | **M** | **ⅢC** | 4+5=9 | **T3** | **N0** | **M0** |
| E06 | Adjacent normal prostate tissue | 72 | **M** | - | - | - | - | - |
| E07 | Adenocarcinoma | 69 | **M** | **ⅢC** | 4+5=9 | **-** | **N0** | **M0** |
| E08 | Adjacent normal prostate tissue | 69 | **M** | - | - | - | - | - |
| E09 | Adenocarcinoma | 60 | **M** | **ⅢC** | 4+5=9 | **-** | **N0** | **M0** |
| E10 | Adjacent normal prostate tissue | 60 | **M** | - | - | - | - | - |
| E11 | Adenocarcinoma | 73 | **M** | **ⅢC** | 4+5=9 | **-** | **N0** | **M0** |
| E12 | Adjacent normal prostate tissue | 73 | **M** | - | - | - | - | - |
| E13 | Adenocarcinoma | 70 | **M** | **ⅢC** | 5+4=9 | **T3** | **N0** | **M0** |
| E14 | Adjacent normal prostate tissue | 70 | **M** | - | - | - | - | - |
| E15 | Adenocarcinoma | 61 | **M** | **ⅢC** | 5+4=9 | **T3** | **N0** | **M0** |
| E16 | Adjacent normal prostate tissue | 61 | **M** | - | - | - | - | - |
| F01 | Adenocarcinoma | 60 | **M** | **ⅢC** | 5+4=9 | **T3** | **N0** | **M0** |
| F02 | Adjacent normal prostate tissue | 60 | **M** | - | - | - | - | - |
| F03 | Adenocarcinoma | 67 | **M** | **ⅢC** | 5+5=10 | **T3** | **N0** | **M0** |
| F04 | Adjacent normal prostate tissue | 67 | **M** | - | - | - | - | - |
| F05 | Adenocarcinoma | 69 | **M** | **ⅢC** | 5+5=10 | **T4** | **N0** | **M0** |
| F06 | Adjacent normal prostate tissue | 69 | **M** | - | - | - | - | - |
| F07 | Adenocarcinoma | 62 | **M** | **ⅣA** | 3+3=6 | **-** | **N1** | **M0** |
| F08 | Adjacent normal prostate tissue | 62 | **M** | - | - | - | - | - |
| F09 | Adenocarcinoma | 78 | **M** | **ⅣA** | 3+4=7 | **-** | **N1** | **M0** |
| F10 | Adjacent normal prostate tissue | 78 | **M** | - | - | - | - | - |
| F11 | Adenocarcinoma | 73 | **M** | **ⅣA** | 3+4=7 | **T3** | **N1** | **M0** |
| F12 | Adjacent normal prostate tissue | 73 | **M** | - | - | - | - | - |
| F13 | Adenocarcinoma | 72 | **M** | **ⅣA** | 4+3=7 | **T3** | **N1** | **M0** |
| F14 | Adjacent normal prostate tissue | 72 | **M** | - | - | - | - | - |
| F15 | Adenocarcinoma | 78 | **M** | **ⅣA** | 4+3=7 | **T3** | **N1** | **M0** |
| F16 | Adjacent normal prostate tissue | 78 | **M** | - | - | - | - | - |
| G01 | Adenocarcinoma | 74 | **M** | **ⅣA** | 3+5=8 | **T4** | **N1** | **M0** |
| G02 | Adjacent normal prostate tissue | 74 | **M** | - | - | - | - | - |
| G03 | Adenocarcinoma | 65 | **M** | **ⅣA** | 5+4=9 | **T3** | **N1** | **M0** |
| G04 | Adjacent normal prostate tissue | 65 | **M** | - | - | - | - | - |
| G05 | Adenocarcinoma | 62 | **M** | **ⅡA** | 3+3=6 | **T2** | **N0** | **M0** |
| G06 | Adenocarcinoma | 72 | **M** | **ⅡB** | 3+4=7 | **T2** | **N0** | **M0** |
| G07 | Adenocarcinoma | 62 | **M** | **ⅡB** | 3+4=7 | **T2** | **N0** | **M0** |
| G08 | Adenocarcinoma | 83 | **M** | **ⅢB** | 3+3=6 | **T3** | **N0** | **M0** |
| G09 | Adenocarcinoma | 76 | **M** | **ⅢB** | 3+4=7 | **T3** | **N0** | **M0** |
| G10 | Adenocarcinoma | 72 | **M** | **ⅢB** | 3+4=7 | **T3** | **N0** | **M0** |
| G11 | Adenocarcinoma | 63 | **M** | **ⅢB** | 3+4=7 | **T3** | **N0** | **M0** |
| G12 | Adenocarcinoma | 70 | **M** | **ⅢB** | 3+4=7 | **T3** | **N0** | **M0** |
| G13 | Adenocarcinoma | 69 | **M** | **ⅢB** | 3+4=7 | **T3** | **N0** | **M0** |
| G14 | Adenocarcinoma | 75 | **M** | **ⅢB** | 3+4=7 | **T3** | **N0** | **M0** |
| G15 | Adenocarcinoma | 76 | **M** | **ⅢB** | 4+3=7 | **T3** | **N0** | **M0** |
| G16 | Adenocarcinoma | 71 | **M** | **ⅢB** | 4+3=7 | **T3** | **N0** | **M0** |
| H01 | Adenocarcinoma | 71 | **M** | **ⅢB** | 4+3=7 | **T3** | **N0** | **M0** |
| H02 | Adenocarcinoma | 76 | **M** | **ⅢB** | 4+3=7 | **T3** | **N0** | **M0** |
| H03 | Adenocarcinoma | 76 | **M** | **ⅢB** | 3+5=8 | **T3** | **N0** | **M0** |
| H04 | Adenocarcinoma | 77 | **M** | **ⅢB** | 4+4=8 | **T4** | **N0** | **M0** |
| H05 | Adenocarcinoma | 65 | **M** | **ⅢB** | 4+4=8 | **T4** | **N0** | **M0** |
| H06 | Adenocarcinoma | 64 | **M** | **ⅢB** | 4+4=8 | **T3** | **N0** | **M0** |
| H07 | Adenocarcinoma | 69 | **M** | **ⅢB** | 4+4=8 | **T3** | **N0** | **M0** |
| H08 | Adenocarcinoma | 71 | **M** | **ⅢB** | 5+3=8 | **T3** | **N0** | **M0** |
| H09 | Adenocarcinoma | 70 | **M** | **ⅢB** | 5+3=8 | **T4** | **N0** | **M0** |
| H10 | Adenocarcinoma | 83 | **M** | **ⅢB** | 5+3=8 | **T3** | **N0** | **M0** |
| H11 | Adenocarcinoma | 68 | **M** | **ⅢB** | 5+3=8 | **T3** | **N0** | **M0** |
| H12 | Adenocarcinoma | 75 | **M** | **ⅢC** | 4+5=9 | **T3** | **N0** | **M0** |
| H13 | Adenocarcinoma | 74 | **M** | **ⅢC** | 4+5=9 | **T3** | **N0** | **M0** |
| H14 | Adenocarcinoma | 63 | **M** | **ⅢC** | 4+5=9 | **T3** | **N0** | **M0** |
| H15 | Adenocarcinoma | 81 | **M** | **ⅢC** | 4+5=9 | **T3** | **N0** | **M0** |
| H16 | Adenocarcinoma | 62 | **M** | **ⅢC** | 4+5=9 | **T3** | **N0** | **M0** |
| I01 | Adenocarcinoma | 75 | **M** | **ⅢC** | 5+4=9 | **T3** | **N0** | **M0** |
| I02 | Adenocarcinoma | 79 | **M** | **ⅢC** | 5+4=9 | **T4** | **N0** | **M0** |
| I03 | Adenocarcinoma | 59 | **M** | **ⅢC** | 5+4=9 | **T3** | **N0** | **M0** |
| I04 | Adenocarcinoma | 57 | **M** | **ⅢC** | 5+4=9 | **T2** | **N0** | **M0** |
| I05 | Adenocarcinoma | 67 | **M** | **ⅢC** | 5+4=9 | **T4** | **N0** | **M0** |
| I06 | Adenocarcinoma | 70 | **M** | **ⅢC** | 5+4=9 | **T3** | **N0** | **M0** |
| I07 | Adenocarcinoma | 74 | **M** | **ⅣA** | 4+3=7 | **T3** | **N1** | **M0** |
| I08 | Adenocarcinoma | 76 | **M** | **ⅣA** | 4+3=7 | **T4** | **N1** | **M0** |
| I09 | Adenocarcinoma | 69 | **M** | **ⅣA** | 4+3=7 | **-** | **N1** | **M0** |
| I10 | Adenocarcinoma | 73 | **M** | **ⅣA** | 4+3=7 | **T3** | **N1** | **M0** |
| I11 | Adenocarcinoma | 62 | **M** | **ⅣA** | 4+3=7 | **T3** | **N1** | **M0** |
| I12 | Adenocarcinoma | 76 | **M** | **ⅣA** | 3+5=8 | **T4** | **N1** | **M0** |
| I13 | Adenocarcinoma | 83 | **M** | **ⅣA** | 4+4=8 | **T3** | **N1** | **M0** |
| I14 | Adenocarcinoma | 83 | **M** | **ⅣA** | 4+4=8 | **T4** | **N1** | **M0** |
| I15 | Adenocarcinoma | 72 | **M** | **ⅣA** | 4+4=8 | **T3** | **N1** | **M0** |
| I16 | Adenocarcinoma | 67 | **M** | **ⅣA** | 4+4=8 | **T3** | **N1** | **M0** |
| J01 | Adenocarcinoma | 71 | **M** | **ⅣA** | 4+5=9 | **T3** | **N1** | **M0** |
| J02 | Adenocarcinoma | 78 | **M** | **ⅣA** | 4+5=9 | **T3** | **N1** | **M0** |
| J03 | Adenocarcinoma | 70 | **M** | **ⅣA** | 4+5=9 | **T3** | **N1** | **M0** |
| J04 | Adenocarcinoma | 63 | **M** | **ⅣA** | 4+5=9 | **T3** | **N1** | **M0** |
| J05 | Adenocarcinoma | 76 | **M** | **ⅣA** | 5+4=9 | **T3** | **N1** | **M0** |
| J06 | Adenocarcinoma | 72 | **M** | **ⅣA** | 5+4=9 | **T3** | **N1** | **M0** |

**Table S2. The H-score of patients for TMA**

| Sites | NAT10 | HMGA1 | KRT8 |
| --- | --- | --- | --- |
| A01 | 62.25 | 92.86 | 147.9 |
| A02 | 95.75 | 71.55 | 57.23 |
| A03 | 91.78 | 79.82 | 36.84 |
| A04 | 84.56 | 33.27 | 49.75 |
| A05 | 127.96 | 99.16 | 78.74 |
| A06 | 45.74 | 68.12 | 46.72 |
| A07 | 128.42 | 96.45 | 108.37 |
| A08 | 122.57 | 37.18 | 46.12 |
| A09 | 28.18 | 53.95 | 98.58 |
| A10 | 23.13 | 30.42 | 41.51 |
| A11 | 63.91 | 82.27 | 41.95 |
| A12 | 82.21 | 71.68 | 41.19 |
| A13 | 34.51 | 11.6 | 0.0325 |
| A14 | 119.54 | 23.04 | 66.04 |
| A15 | 68.53 | 10.23 | 49.85 |
| A16 | 41.35 | 27.29 | 30.83 |
| B01 | 118.45 | 114.7 | 140.14 |
| B02 | 96.35 | 73.67 | 37.14 |
| B03 | 184.73 | 114.35 | 109.02 |
| B04 | 126.66 | 101.34 | 77.32 |
| B05 | 95.63 | 125.3 | 161 |
| B06 | 99.32 | 66.71 | 88.21 |
| B07 | 111.73 | 72.74 | 121.12 |
| B08 | 138.24 | 79.08 | 46.13 |
| B09 | 141.16 | 109.16 | 45.14 |
| B10 | 119.34 | 68.41 | 45.02 |
| B11 | 181.23 | 95.04 | 58.57 |
| B12 | 71.97 | 72.97 | 64.92 |
| B13 | 128.42 | 52.38 | 117.92 |
| B14 | 132.91 | 58.15 | 35.85 |
| B15 | 100.88 | 23.29 | 49.04 |
| B16 | 159.41 | 83.71 | 20.82 |
| C01 | 15.18 | 61.69 | 141.98 |
| C02 | 143.07 | 80.28 | 41.72 |
| C03 | 122.77 | 87.16 | 31.44 |
| C04 | 106.28 | 77.13 | 62.59 |
| C05 | 119.47 | 68.6 | 101.92 |
| C06 | 160.61 | 81.01 | 68.88 |
| C07 | 119.36 | 69.48 | 18.03 |
| C08 | 79.08 | 50.19 | 29.24 |
| C09 | 166.25 | 49.46 | 61.16 |
| C10 | 192.52 | 64.04 | 61.31 |
| C11 | 175.37 | 79.8 | 62.05 |
| C12 | 141.59 | 70.82 | 36.63 |
| C13 | 185.26 | 82.68 | 60.99 |
| C14 | 228.61 | 113.7 | 63.58 |
| C15 | 102.51 | 34.55 | 155.61 |
| C16 | 90.11 | 59.29 | 4.221 |
| D01 | 41.16 | 87.67 | 62.86 |
| D02 | 26.59 | 85.14 | 35.26 |
| D03 | 81.28 | 24.34 | 0.0323 |
| D04 | - | - | - |
| D05 | 92.86 | 65.25 | 65.01 |
| D06 | 115.73 | 63.04 | 18.72 |
| D07 | 116.51 | 81.49 | 52.66 |
| D08 | 158 | 94 | 42.2 |
| D09 | 145.13 | 85.56 | 36.69 |
| D10 | 147.42 | 102.64 | 33.09 |
| D11 | 119.76 | 106.38 | 131.4 |
| D12 | 118.35 | 42.27 | 30.23 |
| D13 | 24.68 | 61.93 | 43.35 |
| D14 | 89.15 | 18.04 | 2.639 |
| D15 | 94.65 | 82.56 | 29.18 |
| D16 | 60.39 | 87.79 | 49.74 |
| E01 | 152.26 | 86.41 | 48.87 |
| E02 | 105.78 | 68.01 | 43.59 |
| E03 | 47.67 | 30.69 | 46.5 |
| E04 | 45.6 | 48.63 | 126.52 |
| E05 | 86.83 | 59.84 | 70.67 |
| E06 | 41.15 | 32.82 | 31.32 |
| E07 | 118.42 | 82.76 | 87.4 |
| E08 | 73.76 | 44.67 | 6.232 |
| E09 | 80.57 | 110.48 | 57.81 |
| E10 | 155.61 | 94.03 | 8.179 |
| E11 | 167.98 | 105.61 | 64.92 |
| E12 | 185.53 | 103.33 | 32.41 |
| E13 | 34.74 | 42.47 | 19.4 |
| E14 | 40.51 | 49.09 | 25.94 |
| E15 | 79.49 | 53.18 | 20.81 |
| E16 | 67.01 | 50.3 | 24.38 |
| F01 | 79 | 93.95 | 87.13 |
| F02 | 67.44 | 75.09 | 58.64 |
| F03 | 214.1 | 86.41 | 175.08 |
| F04 | 54.23 | 26.55 | 12.09 |
| F05 | 137.08 | 87.24 | 73.52 |
| F06 | 100.02 | 86.43 | 45.09 |
| F07 | 70.31 | 98.25 | 132.98 |
| F08 | 99.59 | 61.52 | 42.67 |
| F09 | 122.69 | 86.37 | 48.27 |
| F10 | 116.4 | 86.63 | 42.78 |
| F11 | 186.32 | 71.24 | 19.66 |
| F12 | 63.67 | 17.32 | 0.932 |
| F13 | 183.59 | 73.05 | 104.14 |
| F14 | 132.83 | 100.25 | 43.77 |
| F15 | 87.21 | 47.49 | 0.106 |
| F16 | 89.73 | 84.59 | 29.64 |
| G01 | 61.67 | 31.67 | 7.984 |
| G02 | 121.52 | 86.86 | 42.41 |
| G03 | 144.43 | 112.44 | 81.32 |
| G04 | 42.02 | 5.242 | 0.0229 |
| G05 | 31.43 | 62.54 | 98.16 |
| G06 | 154.73 | 76.37 | 31.32 |
| G07 | 163.23 | 111.44 | 83.95 |
| G08 | 171.92 | 82.1 | 41.24 |
| G09 | 154.17 | 100.21 | 12.56 |
| G10 | 107.28 | 106.98 | 7.961 |
| G11 | 122.94 | 105.36 | 149.62 |
| G12 | 66.72 | 90.59 | 49.76 |
| G13 | 86.36 | 44.66 | 4.843 |
| G14 | 115.09 | 59.73 | 54.49 |
| G15 | 147.77 | 67.54 | 67.6 |
| G16 | 122.53 | 66.44 | 38.36 |
| H01 | - | **-** | - |
| H02 | 109.63 | 57.2 | 21.49 |
| H03 | 94.39 | 47.1 | 105.72 |
| H04 | 166.54 | 163.4 | 75.07 |
| H05 | 102.84 | 111.32 | 68.93 |
| H06 | 118.37 | 122.28 | 111.66 |
| H07 | 133.66 | 59.35 | 52.58 |
| H08 | 98.47 | 70.85 | 20.94 |
| H09 | 131.38 | 62.11 | 30.12 |
| H10 | 127.74 | 140.99 | 158.6 |
| H11 | 135.27 | 85.02 | 57.8 |
| H12 | 168.91 | 111.38 | 129.57 |
| H13 | 153.85 | 72.11 | 7.069 |
| H14 | 171 | 99.48 | 84.59 |
| H15 | 269.03 | 103.49 | 130.64 |
| H16 | 128.39 | 54.28 | 32.13 |
| I01 | 90.25 | 66.82 | 17.8 |
| I02 | 284.51 | 80.16 | 164.59 |
| I03 | 172.44 | 101.01 | 56.76 |
| I04 | **-** | **-** | - |
| I05 | 160.09 | 42.91 | 26.76 |
| I06 | 102.74 | 58.07 | 27.39 |
| I07 | 92.18 | 68.74 | 19.09 |
| I08 | 179.83 | 96.49 | 93.59 |
| I09 | 182.85 | 164.6 | 231.57 |
| I10 | 139.09 | 110.89 | 87.26 |
| I11 | 97.9 | 80.39 | 52.76 |
| I12 | 139.95 | 121.98 | 35.87 |
| I13 | 106.5 | 95.31 | 73.71 |
| I14 | 62.89 | 91.43 | 136.97 |
| I15 | 186.61 | 169.97 | 182.67 |
| I16 | 89.84 | 110.68 | 125.5 |
| J01 | 85.9 | 116.15 | 32.64 |
| J02 | 179.41 | 67.04 | 66.55 |
| J03 | 65.2 | 40.44 | 12.54 |
| J04 | 173.47 | 112 | 72.88 |
| J05 | 128.94 | 77.57 | 3.983 |
| J06 | 124.55 | 84.67 | 60.93 |

**Table S3. The short hairpin RNAs used in this study**

| Plasmids | short hairpin RNAs sequences |
| --- | --- |
| sh-NAT10#1 | 5'-CGGCCTTCAGTGCTGTGGTGTTATATTCAAGAGATATAACACCACAGCACTGAAGGCCGTTTTTT-3' |
| sh-NAT10#2 | 5'-CATCAGGATGTGGTGGGAAGATTTATTCAAGAGATAAATCTTCCCACCACATCCTGATGTTTTTT-3' |
| sh-NAT10#3 | 5'-GCGGTGGCATTTGGGTACTCCAATATTCAAGAGATATTGGAGTACCCAAATGCCACCGCTTTTTT-3' |

**Table S4. The Primers used in this study**

| Genes | Primers sequences |
| --- | --- |
| NAT10 | 5'-GCCTCTTGTAAGAAGTGTCTCG-3'  5'-TCTTTTCAGAGATGCCCTCGAT-3' |
| GAPDH | 5'-CAGTCAGCCGCATCTTCTT-3'  5'-GACAAGCTTCCCGTTCTCAG-3' |
| HMGA1 | 5'-AGCGAAGTGCCAACACCTAAG-3'  5'-TGGTGGTTTTCCGGGTCTTG-3' |
| KRT8 | 5'-CAGAAGTCCTACAAGGTGTCCA-3'  5'-CTCTGGTTGACCGTAACTGCG-3' |
| U6 | 5'-CTCGCTTCGGCAGCACA-3'  5'-AACGCTTCACGAATTTGCGT-3' |

**Table S5. The** **small interfering RNAs used in this study**

|  | small interfering RNAs sequences |
| --- | --- |
| si-HMGA1#1 | 5'-GAGUCGAGCUCGAAGUCCATT-3' |
| si-HMGA1#2 | 5'-GCUCCCCUAACCCUACUUUTT-3' |
| si-HMGA1#3 | 5'-GCCAACACCUAAGAGACCUTT-3' |
| si-KRT8#1 | 5'-GGGCUGAGGCUGAGAGCAUTT-3' |
| si-KRT8#2 | 5'-CUCGAAGCAACAUGGACAATT-3' |
| si-KRT8#3 | 5'-GGGAGGCAUCACCGCAGUUTT-3' |
